# Development of an animated infographic on Permanent Health Education*

**DOI:** 10.1590/1518-8345.3536.3311

**Published:** 2020-07-15

**Authors:** Letícia Lopes Dorneles, Vivian Do Prado Martins, Caroline Silva Morelato, Fernanda Dos Santos Nogueira De Goes, Luciana Mara Monti Fonseca, Rosangela Andrade Aukar De Camargo

**Affiliations:** 1 Universidade de São Paulo, Escola de Enfermagem de Ribeirão Preto, PAHO/OMS Collaborating Centre at the Nursing Research Development, Ribeirão Preto, SP, Brazil.

**Keywords:** Education, Continuing Education, Technology, Animation, Nursing Informatics, Learning, Educação, Educação Continuada, Tecnologia, Animação, Informática em Enfermagem, Aprendizagem, Educación, Educación Contínua, Tecnología, Animación, Informática Aplicada a la Enfermería, Aprendizaje

## Abstract

**Objective:**

to create an animated infographic on the concept of Permanent Health Education according to the National Policy and its main differences compared to continuing education and health education.

**Method:**

a methodological study that analyzed context and knowledge gaps with a literature review and brainstorming; synthesized knowledge into a concept map; built and validated a script; created a didactic design; and produced infographic media. 18 representatives from the Center for Permanent Education and Humanization participated in the stages of context analysis and synthesis of knowledge, and six specialists participated in the validation of the script.

**Results:**

through the combination of texts, images, audios, animations and transitions, the concepts, principles and legal journey of the Permanent Education policy were presented with examples from daily work and, at the end, the difference compared to continuing education and health education is presented. The specialist on the theme evaluated the infographic positively as it has clear information that meet the needs of the target audience; favors learning; and is able to circulate in the scientific environment.

**Conclusion:**

the infographic includes content on Permanent Health Education as it represents the daily work scenario and encourages reflection by the health care workers.

## Abstract (pt)

**Objetivo:**

desenvolver infográfico animado sobre o conceito de Educação Permanente em Saúde de acordo com a Política Nacional e suas principais diferenças com relação à educação continuada e educação em saúde.

**Método:**

estudo metodológico por via de análise de contexto e lacunas de conhecimento com revisão de literatura e *brainstorming*; síntese de conhecimentos em mapa conceitual; construção e validação de roteiro; elaboração de desenho didático e produção da mídia infográfica. Participaram 18 interlocutores de Núcleo de Educação Permanente e Humanização nas etapas de análise do contexto e síntese do conhecimento e seis especialistas na validação do roteiro.

**Resultados:**

por meio de associação de textos, imagens, áudios, animações e transições, foram apresentados os conceitos, princípios e a trajetória legal da política de Educação Permanente, com exemplos do cotidiano do trabalho, destacando-se as diferenças com relação à educação continuada e educação em saúde. Os especialistas no tema avaliaram positivamente o infográfico por possuir informações claras, que atendem as necessidades do público-alvo; favorecer o aprendizado e estar apto para circular no meio científico.

**Conclusão:**

o infográfico contempla conteúdos sobre a Educação Permanente em Saúde ao representar o cenário do cotidiano do trabalho e incentiva a reflexão pelos trabalhadores da saúde.

## Abstract (es)

**Objetivo:**

desarrollar una infografía animada sobre el concepto de Educación Permanente en Salud de acuerdo con la Política Nacional y sus principales diferencias con respecto a la educación continua y a la educación en salud.

**Método:**

estudio metodológico que analizó el contexto y las brechas de conocimiento con revisión de la literatura y *brainstorming*, sintetizó el conocimiento en un mapa conceptual; creó y validó el guión; elaboró el diseño didáctico y produjo los medios infográficos. Participaron 18 interlocutores del Núcleo de Educación Permanente y Humanización en las etapas de análisis del contexto y síntesis de conocimientos y seis especialistas en la validación del guión.

**Resultados:**

mediante la asociación de textos, imágenes, audios, animaciones y transiciones, se presentaron los conceptos, los principios y trayectoria la jurídica de la política de Educación Permanente con ejemplos del trabajo cotidiano y, al final, se expone su diferencia en relación con la educación continua y la educación en salud. Los especialistas en la materia evaluaron positivamente la infografía por contener una información clara que satisface las necesidades del público objetivo: favoreciendo el aprendizaje y ser apta circular en el medio científico.

**Conclusión:**

la infografía contempla contenidos sobre la Educación Permanente en Salud al representar el escenario del trabajo diario; además, incentiva la reflexión de los trabajadores de la salud.

## Introduction

Permanent Health Education (PHE) aims to burst the ethical, technical and scientific knowledge of the health care workers and managers, in order to respond to the demands and social health needs in the work process of the health unit to which they belong^(^ 1 ^)^; these actions have collaboratively mobilized educational institutions and health services, with mutual benefits, which translate into advances in the qualification of the care practice and of the training of human resources in health^(^ 2 ^)^, by building new forms of interaction between them and the population^(^ 1 ^,^ 3 ^)^.

Legally, they were enhanced with the publication of the National Policy of Permanent Health Education (*Política Nacional de Educação Permanente em Saúde*, PNEPS)^(^ 4 ^)^, which presupposes the propositional critical thinking of workers, managers, users, and educational institutions, recognized as the PHE quadrilateral^(^ 5 ^-^ 6 ^)^. The PNPES^(^ 7 ^)^ has an ethical and social commitment to the integrity of health care for people and the community^(^ 8 ^)^ by seeking changes that are in line with policies and programs of the Unified Health System (*Sistema* Único *de Saúde*, SUS). Thus, PHE becomes the motion that qualifies assistance and management and should be incorporated into the daily activities of each health unit, as part of solving their problems, by providing collective reflection and offering instruments to change the work practices^(^ 4 ^-^ 9 ^)^.

However, the dynamism of PHE depends on the political, historical, and social contexts to be materialized^(^ 10 ^)^. Its dissemination has been a historic challenge from the very understanding of its meaning, as its assumptions are unknown; it is often used as a synonym for qualification, training or even confused with Continuing Education (CE) and Health Education (HE), both by the health workers and by the managers^(^ 6 ^)^.

Some health professionals still have an incipient view of PHE, reproducing it as specific activities for the transmission of knowledge with pre-defined themes based exclusively on management needs^(^ 11 ^)^. The lack of understanding on the theme^(^ 11 ^-^ 12 ^)^ and difficulties in the use and management of resources destined to PHE^(^ 13 ^)^ have implications for it use, with limitations of resolving actions that could strengthen the care practices and the flow of care in the Health Care Network (*Rede de Atenção à Saúde*, RAS)^(^ 11 ^-^ 13 ^)^.

One of the main strategies used by the managers to implement PHE is dialog, maintaining an open space for collective reflection^(^ 1 ^)^. It is also believed that, by adding new ways of teaching and learning, such as teaching technologies, the workers will be compelled to learn, which can enhance discussions and possibly contribute to close the gaps in knowledge and facilitate the understanding of the meaning of PHE in and for the work context.

Among the technological resources in teaching that can be used, the animated infographics available online stand out for making different contents accessible to different profiles of people, as they use aesthetic components that seduce and capture attention, with the use of images, audios, texts, moving photos, videos, and animations, all at the same time^(^ 14 ^)^. They have a format for visualizing ideas that convey complex information so that they can be quickly explored^(^ 15 ^)^. Therefore, it is gaining popularity in the media, such as newspapers, magazines, Facebook, Twitter, and YouTube, and also as a teaching resource in the teaching-learning process.

Several studies recognize that the animated infographic is a stimulating resource, potentially useful for education with expressive results^(^ 15 ^-^ 16 ^)^ and that learning through an infographic is 6.5 times greater than compared to the reading of texts^(^ 17 ^)^. Advances in information and communication technologies have created a visual culture of the web, in which images, photos, and videos have become a form of social currency to be shared^(^ 18 ^)^.

A review of Brazilian scientific papers on the implications of the Information and Communication Technology (ICT) in the process of permanent health education indicated the ease of access to the ICT and the role of the participants as its main strengths^(^ 19 ^)^.

Given the above, the objective of this study was to develop an animated infographic on the concept of Permanent Health Education, according to the National Policy, and its main differences compared to both continuing education and health education.

## Method

A methodological descriptive study^(^ 20 ^)^ conducted in the following stages: analysis of the context and of the knowledge gaps; synthesis of the knowledge on the theme; creation, review, and validation of the script; didactic design; and media production^(^ 21 ^-^ 22 ^)^.

To analyze the context and the knowledge gaps (stage 1), scientific articles on the theme were read, focusing on the concept and principles of PHE. In addition, a brainstorming session with representatives from the Center for Permanent Education and Humanization (*Núcleo de Educação Permanente e Humanização*, NEPH) of Ribeirão Preto’s XIII Regional Health Department (*Departamento Regional de Saúde*, DRS) of the state of São Paulo, a project partner, collected ideas to create the media. 18 representatives participated in this stage. Inclusion criteria included: being a health care worker and a representative in the NEPH for at least six months. Everyone was informed about the research objective and agreed to sign the Free and Informed Consent Form.

The brainstorming session^(^ 23 ^)^ took place in 2016 in the headquarters of the DRS-XIII in the city of Ribeirão Preto, lasting 1 hour 30 minutes. It was recorded in digital media and later transcribed. For the brainstorming session, it was decided to present examples of animated infographics to the representatives so that they could have an idea of what the technique to be developed was about. Then, the following question was asked: In your understanding, what should be in the animated infographic so that the health professionals understand what Permanent Health Education is and are able to distinguish its differences with both Continuing Education and Health Education? Thus, we started a space for discussions and reflections on the subject.

In the synthesis of the knowledge on the theme (stage 2), the Concept Map (CM) was used to organize information and was created using the CmapTools^®^program, version 5.05.01 Lite^(^ 24 ^)^.

From the CM, the script was built, reviewed and validated (stage 3), with the definition and description of the contents that would make up the infographic and that should be presented to the target audience; the review and validation took place with six professionals with expertise in the PHE area, in order to verify the scientific and pedagogical quality of the material produced. The specialists evaluated the objectives, structure, presentation, and relevance of the infographic, using an instrument^(^ 25 ^)^ adapted for this purpose. The suggestions from the validation were analyzed and accepted. Finally, the text went through a Portuguese-language proofreader for proper spelling corrections.

With the script in hand, the creation of the didactic design began (stage 4). It is an outline that allows for the visualization of what is expected from the final version of the infographic. The ideas selected by the information analysis were added to those generated creatively by the main researcher. In this stage, the storyboard^(^ 26 ^)^ technique was used to describe the content and to detail the sequence of information, images, animations, colors, background, layout, insertion of audios and additional guidelines in each of the screens, for the production of the media, that is, a low fidelity prototype.

This prototype guided the visual programming work (stage 5), carried out with the assistance of a company specialized in computer animations integrated with the researchers. The following programs were used: Adobe Photoshop CS6^®^ for image processing; Adobe Illustrator^®^ for making drawings; Adobe Flash Professional CS6^®^ to create animations; and Adobe Premiere^®^ for edits.

The time taken for the production of the animated infographic was 11 months and its final version is available on YouTube through the following link: https://www.youtube.com/watch?v=2-E2We4CjdU&t=115s.

The research was approved by the Research Ethics Committee, under Opinion No. 1.435.940 and CAAE No. 51909415.1.0000.5393. All the standards for research with human beings, present in Resolution 466/2012 of the National Health Council of Brazil, were fulfilled.

## Results

**Figure 1:**
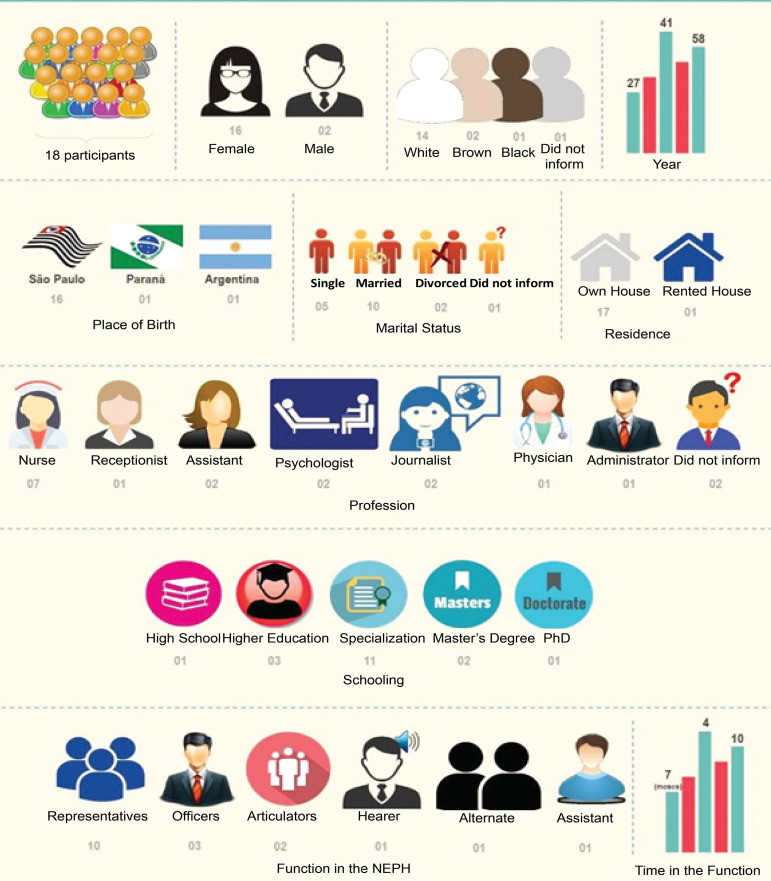
Infographic of the socio-demographic data of the participants in the discussion. Ribeirão Preto, SP, Brazil, 2020

The literature review provided an advanced understanding of the concepts and assumptions of PHE, CE, and HE. Simultaneously to the literature review, with the researchers’ qualified listening on the experiences of the NEPH representatives, it was possible to conduct the brainstorming session. The results on the socio-demographic data of the participants in the brainstorming session, such as gender, age, place of birth, race, marital status, type of household, profession, position at the NEPH, period of employment, form of admission, schooling, and specialization in the area are shown in the infographic in Figure 1.

During the brainstorming session, the participants’ contributions to the infographic focused on how to create its content so that it would relate to the health care workers and really attain the objective of favoring the understanding of the sense and meaning of PHE. The representative’s ideas were added to the script and, later, to the didactic design. Among the points raised, it is possible to highlight some of the situations suggested so that they could be added to the animated infographic, as shown by the statements of the participants below, referred to as “R”, short for representatives: *A theater scenario could be placed with different situations in which Permanent Health Education takes place*(R1)*; Using, as examples, real facts that happen in the daily work*(R2)*; Creating flash pictures of places where Permanent Health Education can happen, like in a discussion group, coffee shop, meeting, patient care, etc.*(R5)*; Giving practical examples of how active methodology can be used in Permanent Health Education*(R9).

**Figure 2:**
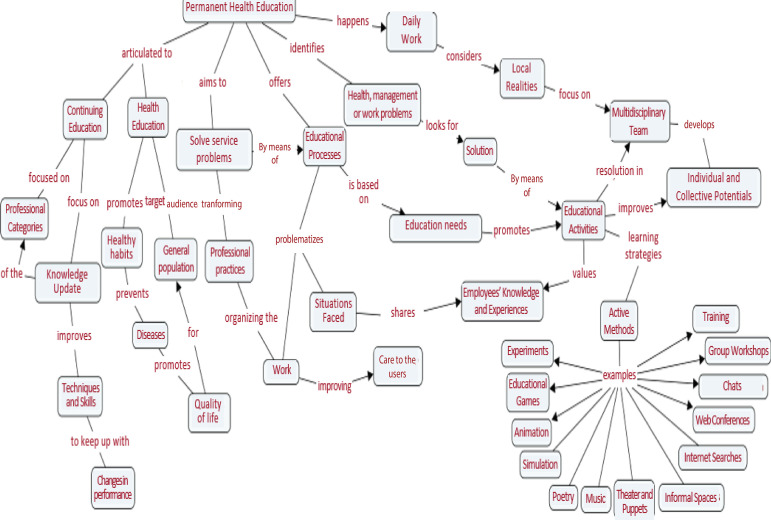
Concept map on permanent health education. Ribeirão Preto, SP, Brazil, 2020

The relevant information from the brainstorming session and from the literature review were ranked and synthesized in a concept map (Figure 2). From the creation of the concept map, the script was consolidated and validated to support the creation of the didactic design.

In the script validation stage, the content of the infographic was evaluated by six specialists in the field using a validation instrument^(^ 25 ^)^. All the items evaluated were classified as adequate or totally adequate, except the item on “Clarity and Objectivity”, which was considered partially adequate in some phrases. The observations made by the specialists were analyzed by the researchers, and the script was modified to meet the suggested corrections.

Through the evaluation and validation of the content, it was possible to identify the relevance of the material, since the specialists evaluated it positively as it has clear, concise information, which meets the needs of the target audience, favors learning in different situations and is able to circulate in the scientific area.

**Figure 3:**
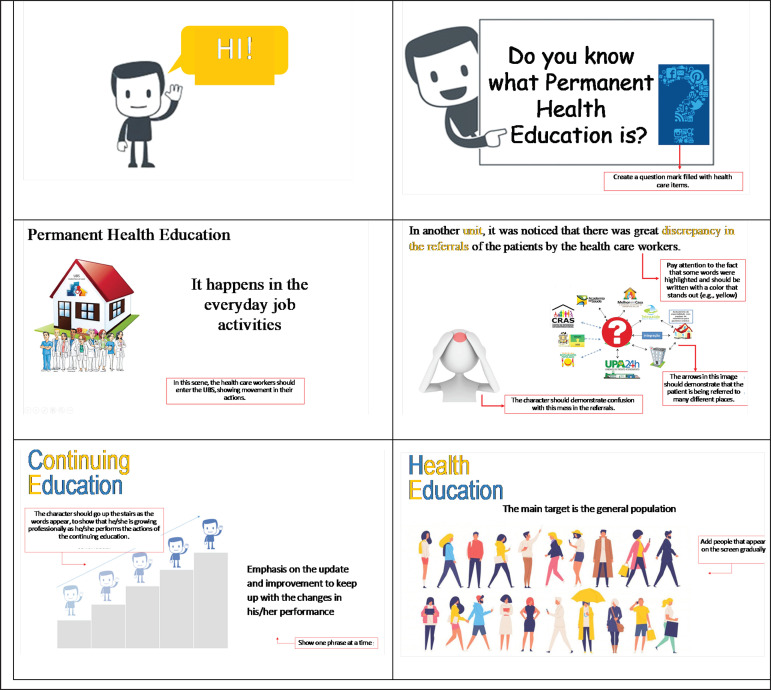
Power Point® screens of the animated infographic prototype. Ribeirão Preto, SP, Brazil, 2020

With the validated and modified content, the didactic design was started using the storyboard technique, by means of Microsoft PowerPoint 2016^®^ in a prototype with each of the screens of the infographic (Figure 3). This prototype was changed seven times during the entire production process, as new ideas emerged or when there was a need to make changes in order to qualify and improve the final version. Altogether, 64 storyboard screens were created in the Power Point^®^presentation. In the process to build the prototype, the researchers used images found in Google Images to exemplify to the programmers what was expected in the infographic, as well as some instructions. However, for media production, the visual programmers created original images.

**Figure 4:**
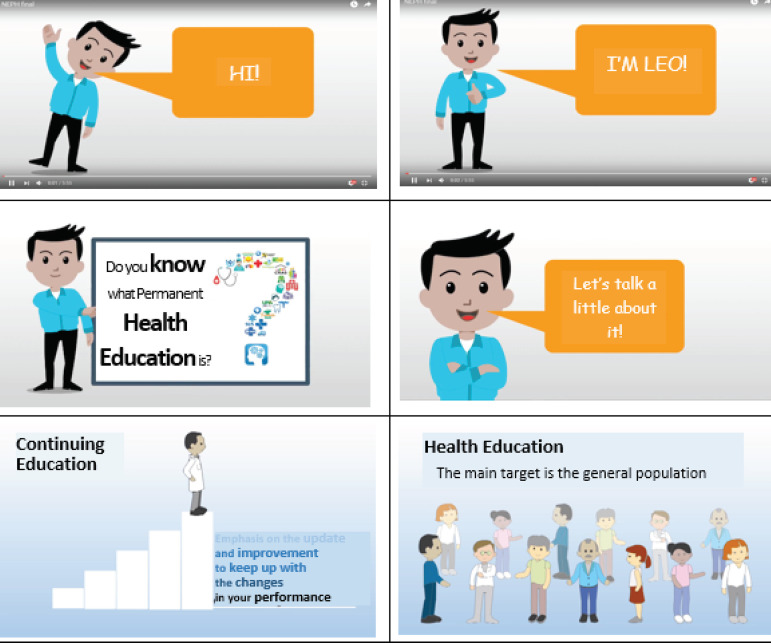
Screens of the finished animated infographic. Ribeirão Preto, SP, Brazil, 2020

With the prototype finished, the animated infographic started to be created by the specialized company. Adobe tools were used, especially Adobe Flash Professional, as they are excellent creation environments to produce different types of interactive and expressive content and because they offer a faithful display of applications, content, and videos through browsers, desktops, laptops, smartphones, tablets, and TVs^(^ 27 ^)^. In Figure 4, it is possible to see some screens of the finished infographic.

An avatar was created as the main character to convey the information to the viewer. Facial and body expressions were added to the character to demonstrate the feelings in each of the scenes. The layout of the images, texts, types and sizes of fonts, colors, animations, transitions, speed of presentation, and highlighting important points were used to make it more aesthetically pleasing, draw attention to the focus of information, and make it easier to receive the information.

Animation and transition effects between the scenes were used to avoid monotony and arouse interest in the viewer. To facilitate the understanding of the content and to instigate questions by the target audience that will watch the animated infographic, simple language with accessible vocabulary was used. A song was also introduced, which extends from the beginning to the end of the presentation to make it more pleasant and dynamic, in order to draw attention and to contribute to the acquisition of the transmitted knowledge. The song chosen was “Blue Skies” by Silent Partner. The choice of this song was due to the fact that it matches the dialog and images presented in the infographic, and because it is a free soundtrack available on YouTube Audio Library^®^. In addition to music, some sounds were also introduced during the infographic to exalt or emphasize certain situations.

## Discussion

Considering the importance of disseminating the PNEPS for advances in the training and qualification of human resources for the SUS, this research aimed to create an animated infographic on the concept of Permanent Health Education according to the PNEPS and of its main differences compared to Continuing Education and to Health Education, since PHE is constantly confused with these other two types of education that are related to the health care workers. This option follows a trend of incorporating new technologies into the educational processes^(^ 19 ^)^, built to meet varied and complex demands in the area of health, in education and in research. Most of the participants in this study are female and work in the field of nursing, but there were also professionals in the areas of psychology, medicine, communication, and administration. It is understood that the diversity of views and knowledge enhanced the collection of information and ideas, considering that the cultural and educational trajectory of each participant broadened the perceptions about reality and how to transform it using the PHE principles. As everyone is a regional NEPH representative and is close to the theme and the dilemmas they experience in their cities^(^ 28 ^)^, the needs were revealed and spontaneously contributed to the guidance of the design by participating in the brainstorming session. The listening of the participants articulated to the knowledge gaps, found in the literature review on the PHE, was visualized in a CM, guiding the production of the script and the didactic design.

In summary, the literature review made it possible to retrieve history on the theme and identified studies carried out on its use in different scenarios. Over the past 30 years, the SUS expanded the coverage of actions and reorganized the health services, which meant an increase in the workforce at various levels of complexity. This scenario^(^ 8 ^,^ 28 ^)^ had implications for the training and qualification of personnel, which started to be strategically articulated by the Ministry of Health’s Department of Labor Management and Health Education in several instances^(^ 29 ^)^. The legal frameworks of PHE, GM/MS Ordinance No. 198/2004 and 1.996/2007, were recently articulated with other SUS policies through the publication of Consolidation Ordinance No. 3/2017 GM/MS^(^ 30 ^)^.

Conceptually, PHE entails “[...] organic relations between teaching and the actions and services, and between teaching and health care, as well as the relations between education and sector management, institutional development and social control in health”^(^ 8 ^)^.

It is aimed at the health team at the various organizational levels of the care network and seeks to qualify the practices for humanizing health care for the population and improving the management of the SUS^(^ 29 ^)^.

Pedagogically, educational actions must start from “[...] problems identified in the work process, using active teaching-learning methodologies, with an emphasis on problem solving, usually through dialogued supervision and workshops, preferably in the work environment itself, in order to raise awareness and to make commitments among the workers, managers, educational institutions, and users regarding the institutional development of the SUS, the improvement of the performance of health teams, and the individual development of the health workers”^(^ 8 ^)^.

However, a number of studies on PHE in different scenarios indicate the difficulty of understanding its assumptions, which can be explained by the historical prevalence in Brazilian education of the Cartesian/traditional method. For the vast majority of the research participants, PHE has a functionalist and technical nature. As spectators of the teaching process, they wait for the transmission of new knowledge that will be learned, in general mechanically, with the purpose of being used at work without a critical reflection of the process^(^ 31 ^-^ 32 ^)^.

Breaking this historical paradigm means adopting the principles of a pedagogy that uses problematization^(^ 33 ^)^, based on the dialogical-dialectic relationship. A conception in which managers, workers, users, and actors of educational institutions reflect on the practice experienced and seek collective solutions for the work process. In this perspective, the focus is on meaningful learning and reflection^(^ 34 ^)^, built from previous knowledge and that makes sense in the daily work. This implies the active participation of the workers in the learning process^(^ 35 ^)^. In this sense, PHE is necessary for the consolidation of a work process that enhances the resolution of the health problems of local populations. The expected result is the democratization of work spaces and progress in improving the quality of health care and humanizing care^(^ 8 ^)^.

Some authors also indicate that, although this policy was created more than 15 years ago, in addition to the difficulty in understanding the meaning of PHE, there is also confusion or ignorance of its definition by professionals and managers, as they often cannot distinguish it from continuing education and/or health education. It is well known that the terms are often used interchangeably in the educational processes. Although some actions have the purpose of permanent education, it is seen that, in practice, they conformed as continuing education or health education, with the confusion persisting regarding the terms, which makes it difficult to organize and implement effective actions aimed at PHE^(^ 36 ^-^ 37 ^)^.

In the daily operations of the health services, the terms “permanent health education”, “continuing education”, and “health education” coexist in the same space. It is important not to consider them conceptually antagonistic in the System, but as educational processes that have different specificities in the teaching-learning process, in which it is necessary to establish their contextualization so that the terms can make sense^(^ 38 ^)^.

Unlike PHE, CE includes activities that have a defined period for execution and uses, more often, the assumptions of traditional teaching methodology. It relates to activities aimed at promoting the sequential and cumulative acquisition of technical and scientific information by the person, in formal educational spaces^(^ 39 ^)^.

On its turn, HE is an educational process aimed at the population, which contributes to increasing the autonomy of people in their care and in the debate with professionals and managers to achieve health care according to their needs^(^ 40 ^)^.

Although the focus of the animated infographic is PHE, the script intended to build a narrative that elucidates, in a clear and accessible language, the main differences between PHE and both CE and HE, and to establish a dialog with practical examples from the reality of work. Therefore, at the beginning of the infographic, the invitation already implies reflection, with questions that follow with explanations supported by the reviewed concepts.

A study in which students of a nursing course created attractive and colorful static infographics using words and visual aids to communicate public health information showed that the task incorporated the use of data and evidence from the literature. In addition, the products were disseminated, serving as vehicles to influence public health and well-being^(^ 41 ^)^.

The common line of design between those productions and the study in question is the optimization of the way of viewing the information that was selected based on evidence in the literature, and which implies planning the presentation of the content of a message, the environment in which it presents itself, and the type of user it is intended for^(^ 42 ^)^.

However, it differs from them by structuring the reviewed information in a CM and by using ideas from the brainstorming session, with the active participation of the representatives in the construction of the design.

This study did not find any research that used CM in the creation of infographics. However, international qualitative studies use this tool to create conceptual or theoretical models based on data^(^ 43 ^-^ 44 ^)^. In Brazil, a literature review with meta-synthesis used this strategy for data extraction and analysis, configuring itself as a multi-modal (semiotics and language) and aesthetics (art and graphic representation) communication^(^ 45 ^)^.

The CM structure was a way for researchers to understand and interpret the PHE phenomenon, by detailing the parts and recognizing the whole. It was an emotional and intellectual investment that created, from a dialectic process, visual data with which the researchers were able to interact in the creation of the script and design.

The brainstorming session contributed ideas to the design; this is a technique that promotes the creativity of different groups, through which thoughts are shared, in order to achieve solutions to practical problems^(^ 46 ^)^. It appeared in the 1950s, and its application was expanded to several areas, including education and research. The literature describes three types of brainstorming*:* verbal or traditional, nominal, and electronic. In verbal or traditional brainstorming (adopted in this study), the members actively participate in dialogs and interactions, sharing their opinions, one at a time, without criticisms, and combining ideas throughout the sessions. In nominal brainstorming, the participants can have ideas individually, without communicating with other members of the group^(^ 47 ^)^. Finally, in electronic brainstorming, the participants use online tools, such as e-mail, chat, and discussion forums to support the discussion process in generating ideas, simultaneously^(^ 48 ^)^.

A literature review^(^ 49 ^)^ on the use of brainstorming in undergraduate studies analyzed 42 studies in different areas of knowledge. The results indicate that dialog helps to overcome cognitive, social, and procedural limitations for a better process to come up with ideas, by stimulating creative thinking, perception, motivation, attention, satisfaction, and understanding of the context, in the search for solutions to the problems.

In this study, the brainstorming session catalyzed the creativity of the representatives by stimulating critical thinking. The proposed challenge contributed to the existing knowledge and to its extent by generating ideas, which most of them were used by the researchers in the making of the infographic.

In this study, the content preceded the design of the infographic or its visual narrative. The infographic is characterized as an expressive type of contemporary view, as it implies the creation of moving images and a density of information^(^ 42 ^)^. Furthermore, the dynamics of this process was supported by the Cognitive Load Theory (CLT), whose principles guided the pedagogical aspects that supported the creation of this educational technology^(^ 50 ^-^ 51 ^)^.

The Cognitive Load translates the load imposed on the cognitive system of people, the result of the Mental Effort involved in carrying out activities or learning new knowledge. CLT’s main focus is on understanding how the limitations of the operational memory, or the basic cognitive structure of the individuals, will influence their ability to manage their mental resources for a particular purpose (learning, for example) when faced with activities that demand more or less of this capacity. CLT acknowledges that cognitive overload is possible when resource constraints are intensified, which can affect cognitive processes in general, such as memory, attention, and perception. Thus, cognitive overload limits the cognitive processing of individuals in carrying out their activities and in learning^(^ 52 ^)^.

Based on the CLT, the reflections pondered the quantity and quality of information or cognitive load, as well as the time spent to clearly expose the concepts that surround the understanding of PHE in its presentation. Therefore, by presenting condensed and measured PHE concepts and principles, the infographic offers the possibility of reflections and deepeningns that may speed up the process of understanding them. However, it dispenses the dialog advocated by Freire’s critical pedagogy in the conversation circles, a *sine qua non* in PHE that supports collective reflection and criticism to understand the cognitive load transmitted by the infographic.

It is believed that the animated infographic can contribute to the learning of workers, without risking a cognitive overload that would imply discouragement and withdrawal from activities. It is worth mentioning that the time allocated to PHE in the health units to gather the health team is scarce, and that an educational technology such as the infographic may possibly disseminate content with greater speed and promote more meaningful meetings.

Furthermore, the validation of the technology by educators improved the quality of the content developed by the researchers, by refining the script and its scientific basis.

Several studies show that the use of the technology enables access to countless learning possibilities, facilitates the understanding of technical and scientific information^(^ 19 ^)^, and increases interest and motivation in the search of knowledge, user satisfaction, and autonomy in learning, with reduced stress to learn^(^ 42 ^,^ 53 ^-^ 55 ^)^. It also favors the combination of theoretical content and practice by promoting different spaces for learning that privilege the (re)construction of knowledge, with more engaging meanings^(^ 43 ^)^.

In PHE, more specifically, in recent years there has been an increase in technological resources to favor educational actions, but still less than the production of traditional didactic teaching resources. It can be said that in Brazil, in general, there is a shortage of computational educational materials as an auxiliary resource to the teaching-learning process, but the panorama has been expanding in recent years^(^ 42 ^)^.

Therefore, it is considered that the animated infographic will allow anyone to access it on the devices they have, such as smartphones, tablets, computers, and laptops, among others, and to adapt it to time and context. The involvement with the issues surrounding PHE and the difference between both CE and HE, in a more dynamic, attractive and pleasant way, translates concepts with practical examples that may favor a democratic reading of the problems of the professional practice, but also with co-responsibility to resolve them.

In this sense, the attention with the set of images, language, sounds, and script provides creativity and originality to the material and, on the other hand, it transmits the idea of earnestness, responsibility, and social commitment; thus, the material has a great potential to contribute to meaningful learning. It is an attractive environment that represents daily work, encourages PHE actions to be developed by the professionals and offers the possibility of being assisted as many times as necessary.

By choosing the animated infographic as an auxiliary educational resource to highlight and problematize the context of the workers of an NEPH, the research involved them in the creation process. The dialog with the participants of the group of professionals who work in the practice of PHE used previous knowledge and experiences and promoted the need to really understand the problems faced in the practice that hinder the understanding on the theme.

It is expected that it will be a teaching technology that not only answers a question, but also motivates the learner to participate in discussions on the theme and raises further questions. And that also offers to the viewer potential for the effectiveness of PHE actions.

It is noteworthy that, although the animated infographic produced for educational purposes has little insertion in the media universe of health, it is considered that the contributions of this study extend to the possibility of expanding a cycle of research on the potential of this technological resource. An initiative that relates to the teaching methods and their strategies, as the animation provides the exposure of information in a responsible way and committed to the context of the topic addressed and its actors, based on the theoretical framework. Finally, the created infographic is a technological innovation that can contribute to the dissemination of PHE, with quick and cheap access, and motivate the search for knowledge by health care workers.

As for the limitations of this study, there is the expensive cost for the creation of an animated infographic. It is understood that this hinders initiatives to create educational technological resources, since funding for the creation of these technologies is often scarce.

## Conclusion

In the expectation of contributing to the dissemination of PHE in Brazil, this methodological study created an animated infographic based on PHE concepts and assumptions, according to the legal framework, and the main differences in relation to Continuing Education and Health Education. The researchers presented the stages of the process and the tools used in the creation of the script and of the design, based on the learning theory, as well as a literature review on the understanding of the theme in the setting of health institutions, with active participation of NEPH representatives.

As it is an innovative technology, a continuous cycle of reflection in action took place, providing significant learning with regard to the research process, linked to the reality of those who will possibly benefit from the produced resource.

It was proven that, according to the specialists’ evaluations, the text of the animated infographic has clear, concise information that meets the needs of the target audience, favors learning in different situations, and is able to circulate in the scientific area. It is understood that these positive evaluations are due to the fact that the creation of the infographic was based on listening to the contributions offered by the representatives, which made it possible for the doubts and concerns on the theme to be addressed in the infographic.

More than just understanding about PHE and its particularities, it is expected to spark new ideas, instill curiosity, and provide reflection on the professional practice, so that the viewer can improve their actions in favor of excellent care to the health needs of the population. Although it was created and thought mainly for health care workers, for offering free access, it is expected that the scientific community, SUS’ patients, students, and the population in general will also benefit from this technological resource.
